# Evolution of Landscape Ecological Risk at the Optimal Scale: A Case Study of the Open Coastal Wetlands in Jiangsu, China

**DOI:** 10.3390/ijerph15081691

**Published:** 2018-08-08

**Authors:** Yongchao Liu, Yongxue Liu, Jialin Li, Wanyun Lu, Xianglin Wei, Chao Sun

**Affiliations:** 1 Department of Geographic Information Science, Nanjing University, Nanjing 210023, China; yongchaoliu@smail.nju.edu.cn (Y.L.); wanyunlu@smail.nju.edu.cn (W.L.); xianglinwei_nju@foxmail.com (X.W.); 2 Key Laboratory of Coastal Zone Exploitation and Protection, Ministry of Land and Resource, Nanjing 210024, China; 3 Collaborative Innovation Center of South China Sea Studies, Nanjing University, Nanjing 210093, China; 4 Jiangsu Provincial Key Laboratory of Geographic Information Science and Technology, Nanjing University, Nanjing 210023, China; 5 Department of Geography & Spatial Information Techniques, Ningbo University, Ningbo 315211, China; lijialin@nbu.edu.cn (J.L.); sunchaonju@yeah.net (C.S.); 6 Institute for East China Sea, Ningbo University, Ningbo 315211, China

**Keywords:** landscape patterns, spatial heterogeneity, landscape ecological risk assessment, remote sensing and GIS, open coastal wetlands

## Abstract

Detailed analysis of the evolution characteristics of landscape ecological risk is crucial for coastal sustainable management and for understanding the potential environmental impacts of a man-made landform landscapes (MMLL). As a typical open coastal wetland, large-scale human activities (e.g., tidal reclamation, fishery activities, wind farm construction, and port construction) have substantially affected the evolution of the coastal ecological environment. Previous landscape ecological risk assessment studies have documented the effectiveness of assessing the quality of ecological environment processes. However, these studies have either focused on the noncoastal zone, or they have not considered the evolution of the spatial characteristics and ecological risk evolution of the landscape at an optimal scale. Here, we present a landscape ecological risk pattern (LERP) evolution model, based on two successive steps: first, we constructed an optimal scale method with an appropriate extent and grain using multi–temporal Landsat TM/OLI images acquired in the years 2000, 2004, 2008, 2013 and 2017, and then we calculated landscape ecological risk indices. Based on this model, the entire process of the spatiotemporal evolution of ecological risk patterns of the open coastal wetlands in Jiangsu, China, was determined. The principal findings are as follows: (1) The main landscape types in the study area are tidal flats and farmland, and the main features of the landscape evolution are a significant increase in aquafarming and a substantial decrease in the tidal flat area, while the landscape heterogeneity increased; (2) In the past 20 years, the areas of low and relatively low ecological risk in the study region were greatly reduced, while the areas of medium, relatively high, and high ecological risk greatly increased; the areas of high-grade ecological risk areas are mainly around Dongtai and Dafeng; (3) The area of ecological risk from low-grade to high-grade occupied 71.75% of the study area during 2000–2017. During the previous periods (2000–2004 and 2004–2008), the areas of low-grade ecological risk were transformed to areas of middle-grade ecological risk area, while during the later periods (2008–2013 and 2013–2017) there was a substantial increase in the proportion of areas of high-grade ecological risk. Our results complement the official database of coastal landscape planning, and provide important information for assessing the potential effects of MMLL processes on coastal environments.

## 1. Introduction

Landscapes have been considered as a system composed of functionally interacting land units [1], which can be characterized based on the diversity and abundance of land cover and landscape pattern metrics [2]. Landscapes are also locations of human activities within which the sustainable use of resources is an important research topic [3], and thus, they are often regarded as a suitable scale for research on the effects of human activities on the environment [4]. Landscape ecology emphasizes the interaction between spatial patterns, ecological processes and scale. Landscape patterns often affect ecological processes (e.g., population dynamics, animal migration, biodiversity and ecological circadian condition) [5,6]. We can obtain a better understanding of ecological processes through the study of spatial patterns, due to the interaction between patterns and processes, and therefore ecological risk analysis based on landscape patterns can provide an integrated assessment of various types of potential ecological impacts and their cumulative consequences [7]. In addition, in landscape ecology, the scale effect refers to the phenomenon in which the spatial heterogeneity of landscape space changes when there are changes in extent or grain [8]. Studies have demonstrated that the spatial resolution (i.e., grain) of the analysis data and the spatial extent of the study area affect landscape analysis results [9]. Hence, to facilitate environmental management and decision making, it is necessary to consider the evolution of the landscape over a sufficiently long time interval.

With increasing influence of human activities on the evolution of the Earth’s surface environment system (ESES), and the overloading of its natural functioning, ESES has entered a stage of physical and chemical evolution within an artificially-driven framework [10]. In particular, since the onset of the Anthropocene [11], numerous man-made landform landscapes (MMLL) have been created via the direct or indirect combination of human activities and natural forces in the context of natural geography [11,12]. Moreover, in many cases, instantaneous information about the factors influencing MMLL processes within a specific region is difficult to obtain, or may not even exist momentarily. Fortunately, satellite remote sensing data may be an excellent substitute [13,14], and advances in satellite remote sensing can provide an unparalleled record of Earth’s status and dynamics [15]. The ever-increasing amount of updated and archived images offers an opportunity to determine the evolution and to assess the impacts of human activities on the eco-environment over large areas.

Ecological risk refers to the probability and possibility of the negative impact under external pressures (e.g., from natural changes or human activities) on ecosystem structure, functioning, and even ecosystem stability and sustainability [16,17], which are reflected in ecosystem health, productivity, and genetic structure, and economic and aesthetic values [18]. Ecological risk assessment is a process that assesses the adverse environmental impacts that may occur or are occurring due to one or more external factors [19]. Landscape ecology emphasizes the interaction between spatial patterns, ecological processes and scale. Landscape patterns often affect ecological processes (e.g., population dynamics, animal migration, biodiversity and ecological circadian conditions) [5,6]. We can obtain a better understanding of ecological processes through the study of spatial patterns, due to the interaction between patterns and processes. Therefore, ecological risk analysis based on landscape patterns can provide an integrated assessment of various types of potential ecological impacts and their cumulative consequences [7]. In landscape transition risk assessment studies, the landscape index used to measure the landscape pattern of an area was often included within the landscape transition risk assessment framework [20]. Several studies have already adopted a more meaningful ecological risk research framework in ecological assessment [21,22]. Specifically, comprehensive knowledge of landscape pattern processes is increasingly important for the quantitative assessment and analysis of the spatial distribution of ecological risk and to determine the evolutionary process of coastal ecosystems [23]. Most previous landscape ecological risk studies have focused on coastal patterns of ecological evolution [24,25,26,27] using land-use data or a range of landscape metrics [27,28,29,30]. However, the use of a landscape transition risk assessment framework is rare in studies of landscape pattern processes.

Human activities are exerting increasing pressure on coastal ecosystems worldwide, which is also true for the open coastal wetlands in Jiangsu, China. Coastal regions are exposed to a wide variety of hazards, such as landscape changes, habitat loss, seawater intrusion, coastal flooding and waterlogging, which are exacerbated by climate change [31,32,33]. Moreover, many marine and coastal projects have been developed to meet human needs [34,35], and the demand for such developments in coastal regions is often met by exploiting natural land (e.g., tidal flats, water, and open spaces), which may considerably reduce the area of ecological land [27]. Importantly, China’s growing economy is increasingly concentrated in coastal regions [36]; thus, the outline of the Jiangsu coastal reclamation development plan (2010–2020) was passed in 2009 [27], and landscapes such as tidal flats, and areas of cordgrass and open seawater are to be used for agriculture and urban development.

Landscape patterns that anthropogenic behaviors interact with represent the spatial consequences of underlying biophysical or socioeconomic processes [37]. At the same time, the evolution of coastal ecological risk patterns is a complicated process and coastal ecosystems are increasingly negatively impacted. Moreover, ecological losses mean that ecosystems in coastal areas are becoming increasingly sensitive and vulnerable under external pressures [38,39]. The recognition of these issues has resulted in increasing research emphasis on coastal dynamics, human development and coastal relationships, reflected by projects such as LOICZ (land-ocean interactions in the coastal zone, International Geosphere-Biosphere Programme core research program) and the FEC (Future Earth-Coasts, a project to support sustainability and adaptation to global change in the coastal zone; https://www.future earthcoasts.org/) [40]. Consequently, in the context of disaster risk reduction, the assessment of landscape features and the evolution of ecological risks are increasingly important for sustainable coastal planning and management, and it also contributes to the growing number of case studies of coastal changes worldwide.

In light of the above, the specific objectives of the present study of the open coastal wetlands in Jiangsu Province (OCWJ) were: (1) to construct landscape ecological risk indices using geographic information systems (GIS), landscape ecological metrics, Landsat-5 Thematic Mapper (TM) and Landsat-8 Operational Land Imagery (OLI) data; (2) to develop evolution models of landscape ecological risk pattern (LERP); and (3) to identify the spatiotemporal variation of landscape ecological risk patterns during the period of 2000–2017.

## 2. Materials and Methods

### 2.1. Study Area

**Figure 1:**
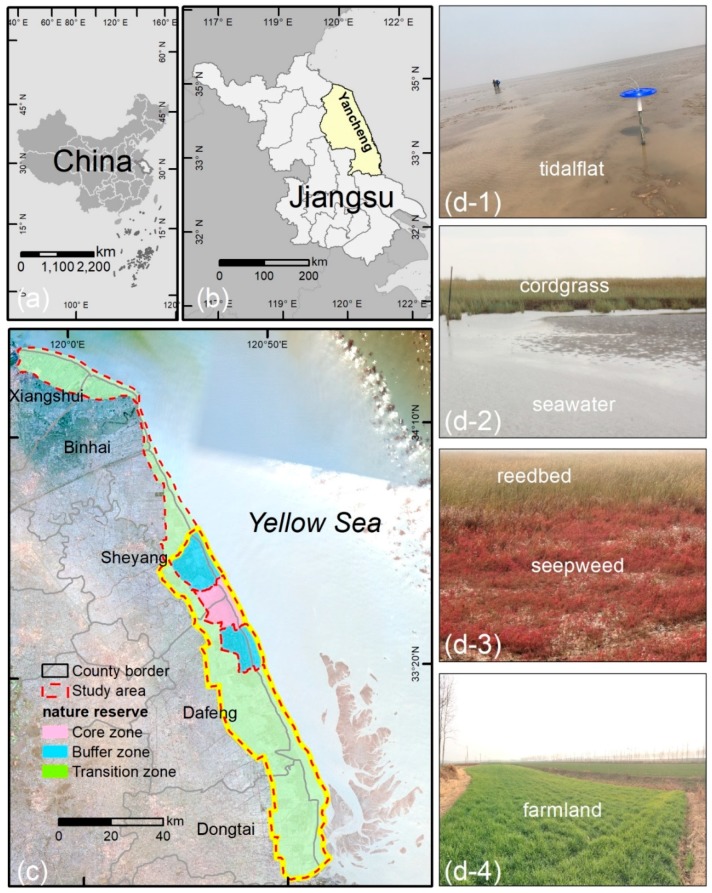
(**a**–**c**) Location of the study area. The main natural wetlands of the open coastal wetlands in Jiangsu (OCWJ) comprise tidalflat (**d-1**); cordgrass and seawater (**d-2**); seepweed and reedbed (**d-3**); and farmland (**d-4**) [42], an exotic introduction from North America which outcompeted the native wetlands and became a dominant species after 1990. The area bounded by the yellow line (**c**) is the spatial heterogeneity of landscape space.

The open coastal wetlands in Jiangsu (OCWJ) lie within the area bounded by 32°34′ and 34°28′ N, and 119°48′ and 120°56′ E (Figure 1). The wetlands were formed by sediment deposition within the old Yellow River Delta and the Yangtze River Delta under the impact of waves and tides in the Yellow Sea and the East China Sea. The study area is affected by the alternation of Tropical Ocean and polar continental air masses governed by the East Asian monsoon. The coastal zone has a maritime monsoon climate ranging from warm-temperate to northern subtropical [41]. The OCWJ are an important pathway and stopover of migratory birds in Northeast Asia–Australia. The terrestrial area of the province encompasses 101,800 km^2^ and it is one of the smallest provinces in China (only 1.06% of the total area). However, the province possesses an extensive coastal area and includes seven coastal cities (e.g., Xiangshui, Binhai, Sheyang, Dafeng, and Dongtai) (Figure 1). In this study, the scope of the Yancheng Wetland Biological Reserve (i.e., the core zone, the buffer zone, and the transition zone, China, http://sts.mep.gov.cn/zrbhq/) was taken as the study boundary.

### 2.2. Data Sets and Processing

Geographic information systems (GIS) and remote sensing (RS), as a mature Earth observation (EO) techniques, are powerful and cost-effective tools for studying the spatiotemporal dynamics of ecological and geographical information [43,44,45]. Landscape data were obtained from Thematic Mapper(TM), and Operational Land Imager(OLI) satellite images in the years 2000, 2004, 2008, 2013, and 2017 (Table 1) from the United States Geological Survey (USGS, http://earthexplorer.usgs.gov/). A total of 10 images were acquired in autumn and winter (October to February) to reflect the true wintering environments of the OCWJ. All the image dataset projections are the Universal Transverse Mercator (UTM) map projection system zone 50N and datum of the World Geodetic System 1984 (WGS84), ensuring consistency between datasets during analysis. Landsat TM/OLI image processing was done using ENVI (version 5.2, the Environment for Visualizing Images, Exiles Visual Information Solutions Company, Virginia, VA, USA.), including image geometric correction, atmospheric correction, image mosaicking, and masking-out the study area. The starting point for this study, especially evident since 2000, was the initial period of unprecedented socioeconomic transformation and intensified human activity which have dramatically modified ecosystems and landscapes across the nation [46]. Auxiliary data consisted of the administrative map of Jiangsu province, a land use map and land use history data, the topographic map of Jiangsu Province (1:50,000), and GPS field survey data and Google Earth samples.

### 2.3. Methodology

**Figure 2:**
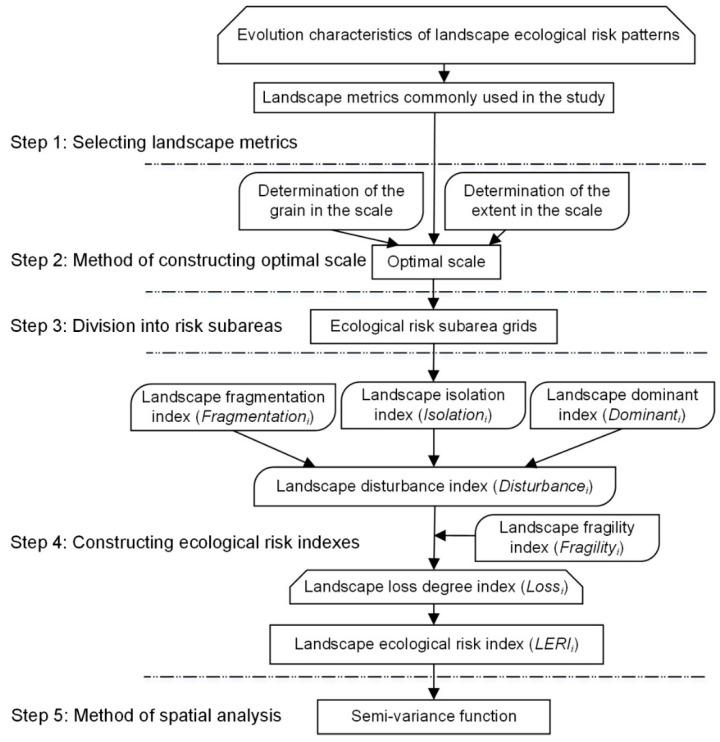
Framework of the methodology used for the landscape ecological risk assessment.

**Figure 3:**
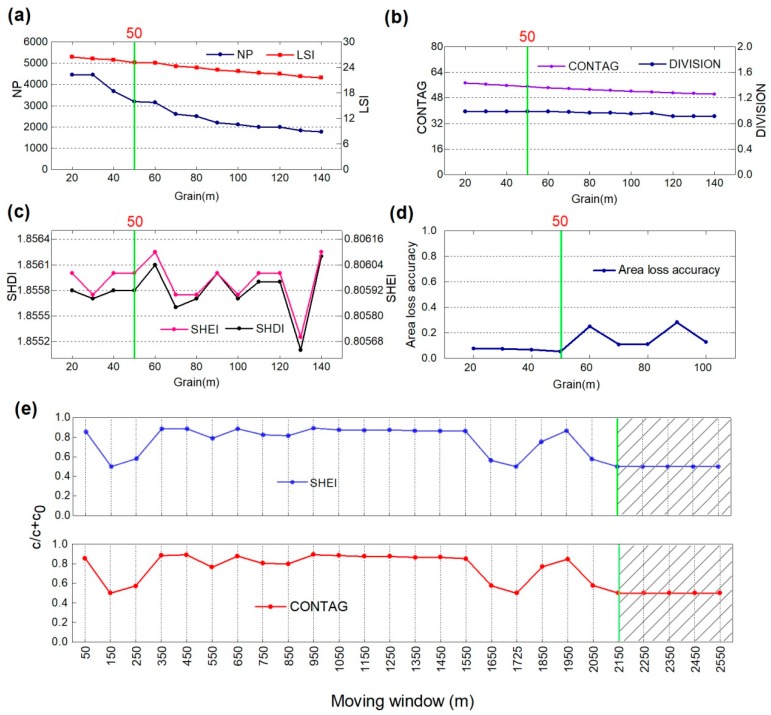
Changes of landscape metric values with grain (**a**–**c**) and changes in area landscape accuracy with different grain (**d**). With the increase in grain size, due to the boundary of the vector data and the changes of the properties of adjacent patches, scale turning points appear in the values of landscape metrics. Considering the distribution of inflection points and the calculation efficiency (**a**–**c**), 20–100 m was selected as the appropriate scale domain to determine the analysis grain for the next step (**d**). The block ratio of different moving windows is obtained (**e**).

A framework was developed for assessing the ecological risks for open coastal wetlands in Jiangsu Province using evolution models of landscape ecological risk pattern (LERP). Figure 2 presents a structural illustration of the methodology applied to ecological risk assessment of the landscape. The structure was formed using a combination of risk assessment techniques—(version 4.2, Oregon State University, Corvallis, OR, USA, available online: http://www.umass.e du/landeco/research/fragstats/fragstats.html) and ArcGIS (version 10.3, ESRI, Redlands, CA, USA, available online: http://www.esri.com). The method involves the following steps:

(1) Step 1: Selecting landscape metrics. The landscape structure in our study area was analyzed using both class-level metrics (each patch type in the given mosaic) and landscape-level metrics (the landscape mosaic as a whole) to investigate and explore landscape scale variables. In addition, reference to previous publications [47,48,49] suggested key landscape metrics (LM) for the assessment of spatial patterns. Since no single LM can be considered [1] for its relevance to structural analysis, we computed 9 class-, 2 landscape-, and 2 class/landscape-level metrics for each map using FRAGSTATS (Table 2). A detailed technical description of each metric is provided for reference in FRAGSTATS [50].

(2) Step 2: Method of constructing optimal scale. Analysis of the grain effect is based on the grain size effect curve of the landscape metrics (LMs) to select the appropriate grain domain [51]. The curve is a horizontal axis with a different grain, and the corresponding LM value is determined on the vertical axis at this grain. Assessment of total area loss in the study area was based on regional land area change evaluation index models [8,52], as expressed in Equations (1) and (2). The conversion grain of 20 m was selected as the starting point, and 140 m as the end point, with an interval of 10 m, and the interpreted vector data were rasterized to obtain a grid map with different grains (Figure 3a–c). According to the area information conservation evaluation (AICE) method [52], using the grain as the abscissa and the regional landscape area change value as the ordinate, the corresponding landscape area change index was calculated separately (Figure 3d). It can be observed that when the grain is less than 50 m, the area loss accuracy value is smaller, and when the grain size is greater than 50 m, the index of area precision loss increases. Consequently, the best analysis grain (50 m) was chosen, which maximizes both the calculation reliability and efficiency.

(1) $$L_{i}=(A_{i}-A_{bi})/A_{bi}\times 100$$

(2) $$S_{i}=\sqrt{\frac{\sum_{i=1}^{n}L_{i}^{2}}{n}}$$

Here, *A_i_* is *i*-type landscape grid area; *A_bi_* is *bi*-type landscape vector area before scale conversion; *L_i_* denotes is the relative loss of area; *S_i_* is the area change index; and *n* is the number of landscape types.

First, based on the analysis of 50-m grain sizes, 26 moving windows of different sizes were obtained using a 50-m interval (i.e., 50, 150, 250 …, 2350, 2450, and 2550 m). Second, moving from the upper left corner of the study area, one grid at a time to the right, the value of the selected landscape metrics in the unit window was calculated, and then the landscape metrics value was assigned to the center grid of the pane, finally producing the relevant LM grid. Third, 5000 random points were generated using ArcGIS 10.3 for the open coastal wetlands in Jiangsu, and the value of the landscape metrics (Shannon’s evenness index, SHEI and the contagion index, CONTAG) representing the landscape was selected. The value of the landscape metrics of each point was imported into GS+ (version 7.0, Geostatistics for the Environmental Sciences, Gamma Design Software, Michigan, USA), and the block ratio of different moving windows was obtained (Figure 3e). It can be seen that when the radius of the moving window is less than 2150 m, the block ratio of the SHEI and CONTAG is erratic and irregular; and when the moving window radius is larger than or equal to 2150 m, the block ratio of the SHEI and CONTAG remains stable and tends to a certain value. Therefore, the selection of 2150 m was the characteristic scale used in this study.

(3) Step 3: Division into risk subareas. To realize the spatialization of landscape ecological risk index (ERI), based on previous research [53], the OCWJ were divided into subareas with a cell size of 7.29 × 7.29 km using an equidistance sampling method which resulted in 106 grid cells (risk subareas). Then, the LERI of each risk grid was calculated and used as the centroid value of each specific ecological risk subarea gird.

(4) Step 4: Constructing ecological risk indexes. This study introduces *Disturbance_i_*, *Fragility_i_*, and *Loss_i_* to construct a landscape ecological risk index. *Disturbance_i_* is used to reflect the extent to which ecosystems represented by different landscapes are disturbed [20], as expressed in Equation 3. *Fragility_i_* indicates the vulnerability of the ecosystem structure within different landscape types (i.e., the higher the index, the more vulnerable the ecosystem). Based on the characteristics of the OCWJ and previous studies [20,54], and previous studies [20,54] and the vulnerability of different landscape types, *Fragility_i_* was first divided into ten levels, from low to high (building, reedbed, seepweed, cordgrass, saltwork, farmland, seawater, aquafarm, tidalflat, and dry pool), and *Fragility_i_* was obtained by normalization processing (Table 3). *Loss_i_* reflects the degree of loss of natural attributes of different landscape types when subjected to external disturbances; it consists of *Disturbance_i_* and *Fragility_i_* [55], as expressed in Equation (7). *LERI_i_* was constructed based on *Loss_i_* in a sample plot so that the landscape spatial pattern was transformed to a spatialized ecological risk variable using the sampling method, as expressed in Equation (8).

(3) $${Disturbance}_{i}=a\times {Fragmentation}_{i}+b\times {Isolation}_{i}+c\times {Do\min ant}_{i}$$

Here, *a*, *b* and *c* are the weights of indices, set to 0.5, 0.3 and 0.2 according to their importance [20].

(4) $${Fragmentation}_{i}=\frac{{NL}_{i}}{{TA}_{i}}$$

Here, *NL_i_* is the number for the *i*th landscape type, and *TA_i_* is the total area of the *i*th landscape type.

(5) $${Isolation}_{i}=\frac{TA}{2{AL}_{i}}\sqrt{\frac{{NP}_{i}}{TA}}$$

Here, *TA* is the landscape total area; *AL_i_* is the area of *i*th land type; and *NP_i_* is the number of patches of the *i*th land type.

(6) $${Do\min ant}_{i}=\frac{\left(PD_{i}+{PF}_{i}\right)}{4}+\frac{{PR}_{i}}{2}$$

Here, *PD_i_* is the patch density (i.e., the number of patches of the *i*th landscape type divided by the total number of patches); *PF_i_* is the patch frequency (i.e., the number of samples of the *i*th landscape type divided by the total number of samples); and *PR_i_* is the patch ratio (i.e., the area of samples of the *i*th landscape type be divided by total area of samples).

(7) $${Loss}_{i}={Disturbance}_{i}\times {Fragility}_{i}$$

(8) $${LERI}_{i}=\sum_{j=1}^{N}\frac{{LAR}_{kj}}{{AR}_{k}}{Loss}_{j}$$

Here, *Loss_i_* is the *i*th landscape loss degree index; *Disturbance_i_* is the *i*th landscape disturbance index; *Fragility_i_* is the landscape fragility index of the *i*th landscape type; *LERI_i_* is the *i*th subarea’s ecological risk index; *Loss_j_* is the *j*th landscape loss degree index; *LAR_kj_* is the *j*th landscape’s area in the *k*th region; and *AR_k_* is an area of the *k*th region.

(5) Step 5: Method of spatial analysis. Geostatistical methods are statistical methods for detecting, simulating and estimating the correlation and distribution of variables within a particular area of study [5]. With the aid of the semi-variance function in geostatistical methods [20,52] it is used to fit the data of different models’ sampling points, and the spatial distribution map of landscape ecological risk index is plotted using the by kriging method. The natural breaks method was used to compare the changes in ecological risk for different periods in the OCWJ. The risks can be classified into categories such as “extremely low (*LERI_i_* < 130.10)”, “low (130.10 ≤ *LERI_i_* < 203.94)”, “relatively low (203.94 ≤ *LERI_i_* < 262.45)”, “medium (262.45 ≤ *LERI_i_* < 294.49)”, “relatively high (294.49 ≤ *LERI_i_* < 330.71)”, “high (330.71 ≤ *LERI_i_* < 372.50)”, and “extremely high (*LERI_i_* ≥ 372.50). On this basis, the distribution map of ecological risk in OCWJ can be used to assess the regional ecological risk.

## 3. Results

### 3.1. Landscape Classification and Landscape Pattern Mapping

**Figure 4:**
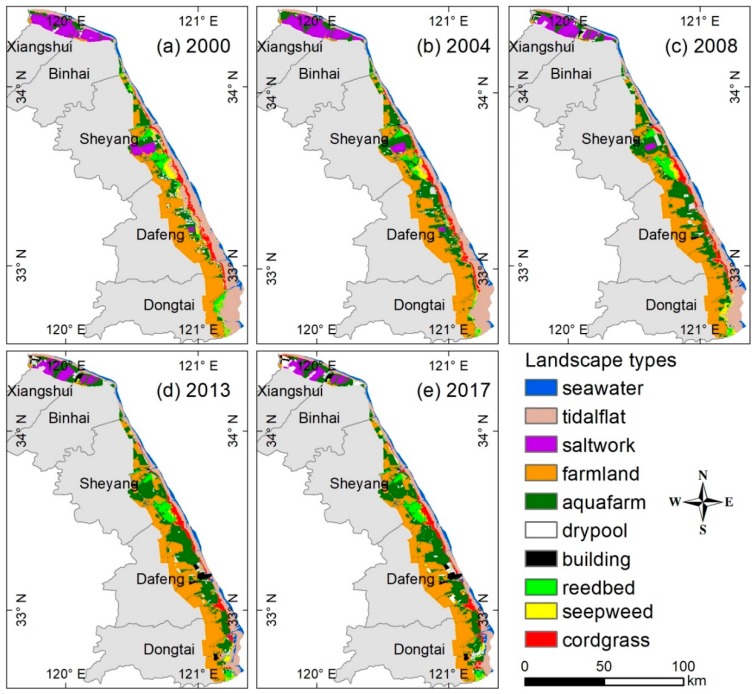
Landscape classification during five periods from 2000–2017 in the open coastal wetlands in Jiangsu, China. (**a**) Map of landscape types in the year 2000; (**b**) map of landscape types in the year 2004; (**c**) map of landscape types in the year 2008; (**d**) map of landscape types in the year 2013; (**e**) map of landscape types in the year 2017.

Ten landscape classes were identified considering the specific local conditions, the national land resources classification system, and following the approach of Xu [27]. These landscape types were modified and verified using ENVI5.2 and ArcGIS10.3. The overall accuracy of each classified image is around 87%, which was verified using field surveys and Google Earth samples. The overall accuracies were within the acceptable range for further analysis [27,56]. The areal extent of ten landscape types (seawater, tidal flat, salt work, farmland, aquafarm, dry pool, building, reedbed, seepweed, and cordgrass) and their spatial distribution, for each time step, are presented in Figure 4. After classification, ten landscape types in different periods were treated as the OCWJ ecological risk receptors and the ecological risk indexes were constructed for them also. Finally, with these available spatial data sets and spatial analysis tools and techniques, the characteristics of a model of the evolution of landscape ecological risk patterns (LERP) of the OCWJ were investigated.

### 3.2. Landscape Pattern Analysis Using an Optinal Scale 

With the intensification of human activities, the landscape pattern, as expressed by various landscape metrics of the individual landscape types of the coastal ecosystems they constitute, changed substantially during 2000–2017 (Figure 4). In 2000, tidal flat, farmland, and aquafarms were the main landscape types in the study area, accounting for 68.75% of the total area, followed by salt works, seawater, dry pool, reedbed, cordgrass, and seepweed. The area of tidalflat was reduced by 458.82 km^2^ during 2000–2017, with the most significant decrease during 2000–2004 (by 132.11 km^2^) and during 2008–2013 (by 152.32 km^2^). Most of the tidal flat area is occupied by aquafarms and farmland. From 2000 to 2017, the area of aquafarms increased by 479.88 km^2^ (with the largest increase of 14.10%). Evidently, therefore, besides natural factors, individual or artificial management activities were effective agents of landscape change in the study area.

**Figure 5:**
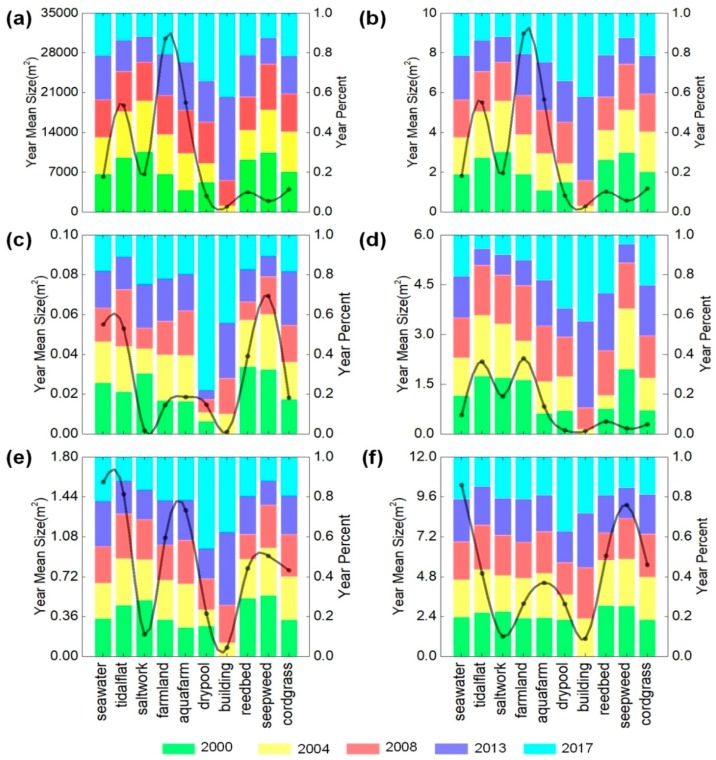
Overall characteristics of landscape types. (**a**) CA; (**b**) PLAND; (**c**) PD; (**d**) LPI; (**e**) ED; and (**f**) LSI. To fully reflect the characteristics of the landscape pattern and to reduce the redundancy of the pattern information, the landscape metrics at landscape level were selected, and the characteristics of the landscape pattern were analyzed using the FRAGSTATS 4.2 selection method.

Changes in different landscape types in the study area are also reflected in the landscape pattern metrics (Figure 5). Year mean size (YMS) and year percent (YP) values of the metrics used to define landscape pattern in the study area were calculated based on the standard method, using FRAGSTATS 4.2. Changes in the trend of YMS of the total area (CA), the percent of the landscape (PLAND), and the largest patch index (LPI), are consistent within 2000–2017, indicating the continued dominance of farmland and tidal flats. The patch density (PD) of the man-made landforms of the landscape remained low within the landscape types, indicating the encroachment upon and dramatic fragmentation of the non-man-made landforms landscape. The year mean size (YMS) of the PD, the edge density (ED), and the landscape shape index (LSI) exhibited similar trends, but there are differences in the amplitudes of the peaks. Patch density (PD) and landscape shape index (LSI) were highest in seepweed along the landscape transfer direction, and ED was highest in seawater. In the landscape class percent of the study area, the class year percent (YP) of saltworks in the CA decreased from 30.35% to 11.85% during 2000–2017, while the buildings area increased from zero to 42.06% in 2017. Moreover, the class year percent (YP) of ED and LSI of the landscape types was increasing for seawater, farmland, dry pool, and buildings landscapes, the most notable being buildings and dry pools. In the last 20 years, the class year percent (YP) of ED of the buildings area increased by 37.74%, and the landscape shape index (LSI) of the drypool increased by 40%. This shows that the continuous development of the study area during 2000–2017 and the continuous expansion of the built area have tended to render the landscape pattern of the area more fragmented; in addition, the boundary between buildings and other landscapes is more complex.

**Figure 6:**
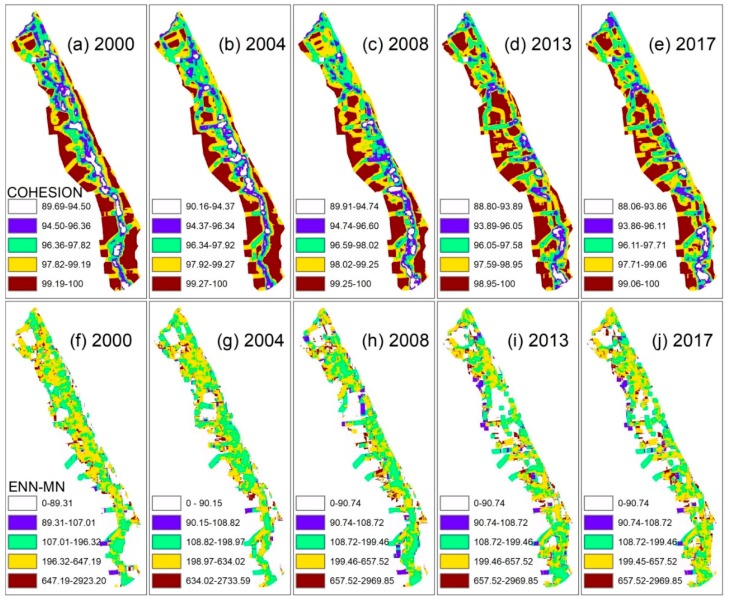
(**a**–**e**) Comparison of the landscape heterogeneity of COHESION during five intervals from 2000–2017. The degree of mosaicism of the landscape pattern is represented by the degree of COHESION. The greater the COHESION value, the higher the degree of patch connectivity of the landscape and the lower the degree of spatial mosaic; (**f**–**j**) Comparison of the landscape heterogeneity of ENN–MN during five intervals from 2000–2017. Distance analysis of the landscape is expressed by ENN–MN. The larger the value of ENN–MN, the greater the distance between the landscapes type blocks and the more scattered the distribution.

Heterogeneity, an inherent landscape attribute, is defined as the complexity and/or variability of a system property in space and/or time [5]. To further analyze the heterogeneity of the landscape, the central and southern regions, with a wide range of landscape categories, were selected for analysis (Figure 6). Using GIS technology, the raster data of 50 m was imported into FRAGSTATS 4.2, and the spatial range of the patch cohesion index (COHESION) and the Euclidean nearest neighbor distance mean index (ENN–MN) visualization were implemented with a moving window of 2150 m. The COHESION in the feature scale space is 88.06–100 (i.e., the degree of tessellation is from low to high) (Figure 6a–e). The main landscape types in the low-value areas of the open coastal wetlands in the Jiangsu (COWJ) landscape mosaic are farmlands, reedbeds, and aquafarms, where farmland and aquafarms are man-made landform landscapes (MMLLs). High-value areas are distributed on the landside of the study area and exhibit a north–south-aligned striped pattern.

From 2000–2004, the ENN–MN decreased within the entire study area (especially on the landward side of Sheyang and Xiangshui), indicating the diffusion of human activity (Figure 6f–j). This was mainly due to the addition of large man-made landform landscapes development units (i.e., salt works). However, from 2004–2008, the southeastern parts of the Sheyang and Dafeng areas exhibited coalescence characteristics in ENN–MN, while the western rural areas of Dafeng areas exhibited a diffusion mode (mainly farmlands and aquafarms). From 2008–2017, the entire center of the study area was again in coalescence mode, perhaps because of the renewed expansion of human activity beyond its original boundaries. Therefore, the diffusion–coalescence process in the OCWJ is unidirectional in time, but is an iterative process. It can be observed that the heterogeneity of the landscape in the OCWJ region was largely caused by human disturbance and the resulting impacts on the coastal wetland ecosystem after the landscape was modified.

### 3.3. Spatiotemporal Differences in Landscape Ecological Risk Patterns

**Figure 7:**
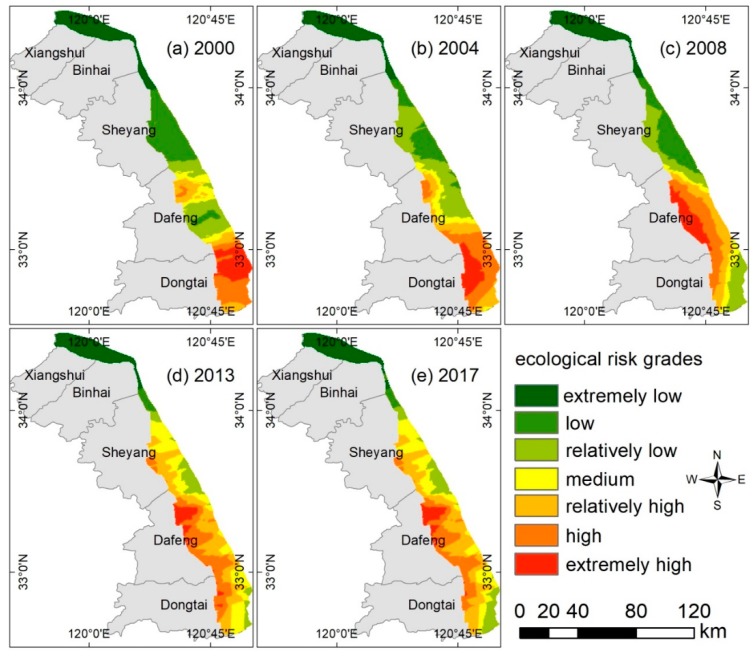
Evolution of the spatial distribution of ecological risk index grades of the open coastal wetlands in Jiangsu (OCWJ) during the five periods from 2000–2017. (**a**) Map of the spatial distribution of ecological risk index grades in the year 2000; (**b**) map of the spatial distribution of ecological risk index grades in the year 2004; (**c**) map of the spatial distribution of ecological risk index grades in the year 2008; (**d**) map of the spatial distribution of ecological risk index grades in the year 2013; (**e**) map of the spatial distribution of ecological risk index grades in the year 2017.

**Figure 8:**
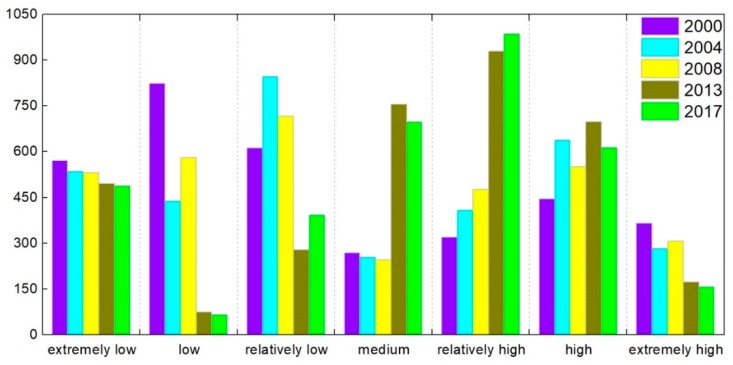
Changes in the areas of different ecological risk grades from 2000–2017. Based on the analysis of the ecological risk level of open coastal wetlands in Jiangsu, the area occupied by the ecological risk grades of 106 risk communities in the study area were analyzed statistically.

The numbers of patches and areas of all landscape types in 2000, 2004, 2008, 2013, and 2017 were calculated using ArcGIS10.3 and FRAGSTATS 4.2. Using Equations (3)–(7), the landscape pattern indexes for all landscape types were calculated and summarized in the OCWJ for the five years (Table 3). Maps of landscape types are shown in Figure 7, and they demonstrate that the landscape ecological risk of the OCWJ changed substantially during 2000–2017. The specific statistics for the different risks grades are shown in Figure 8.

In terms of spatial characteristics, there are two main centers of extreme values in the areas of high-grade ecological risk, namely the offshore area of Dongtai in southern Jiangsu and the landward side of Dafeng. The level of ecological risk in the southern region of the open coastal wetlands in Jiangsu (OCWJ) is significantly higher than that of elsewhere. The intensity of human activity between Dafeng and Dongtai gradually increased, resulting in a more frequent succession of landscape types. The landscape types in the northern region of the OCWJ are relatively few, with saltwork as the main areas, accompanied by farmland. The process of development of the man-made landform landscapes (MMLL) was gradual. The disturbance of the landscape by human activities was less in the southeast, and thus the ecological risk level is relatively low. The centers of high values in the central and southern regions are near Dafeng and Dongtai, and the main landscape types are farmland, fish ponds, tidal flats, and construction land. Owing to the construction of MMLL and the intensive development of farmland and tidal flats, the ecological risk value rises. There is a trend of increasing ecological risk on the landward side of areas adjacent to the sea; the main reason for this is the continual movement of cofferdams towards the sea. Overall, diverse human activities have increased the MMLL and decreased the extent of natural landscapes, with a corresponding increase in ecological risk.

In general, in the areas of economic activity and human habitation, the areas with a high degree of landscape transformation also have high levels of ecological risk, while single landscape types have only a low level of ecological risk. The high-grade ecological risk zone remained dominant in the southern part and its area gradually increased. The low-grade (i.e., extremely low, low, and relatively low) ecological risk areas are distributed around the areas of medium- and high-grade ecological risk (i.e., relatively high, high, extremely high) and they generally expanded in a circular pattern. This shows that there was a diverse pattern of the formation of man-made landform landscapes (MMLL) following reclamation of the coastal wetland, landscape fragmentation was intensified, and the average patch area of natural landscapes such as seepweed was greatly reduced. At the same time, the reduction in the tidal flat area also impacted the coastal ecosystem. Therefore, it is important to pay attention to wetland protection during the development and utilization of open coastal wetlands in Jiangsu (OCWJ).

In the time series, the low-grade ecological risk area of the OCWJ decreased by 1058.55 km^2^ during 2000–2017. The area of extremely low ecological risk was reduced by 82.38 km^2^; the area of low ecological risk was reduced by 757.06 km^2^; and the area of relatively low ecological risk was reduced by 219.12 km^2^. Medium and relatively high are the dominant types of ecological risk in the study area, accounting for 20.51% and 28.99% of the total area in 2017, respectively. At the same time, the area of high ecological risk area increased by 168.88 km^2^.

### 3.4. Landscape Ecological Risk Transfer Analysis

**Figure 9:**
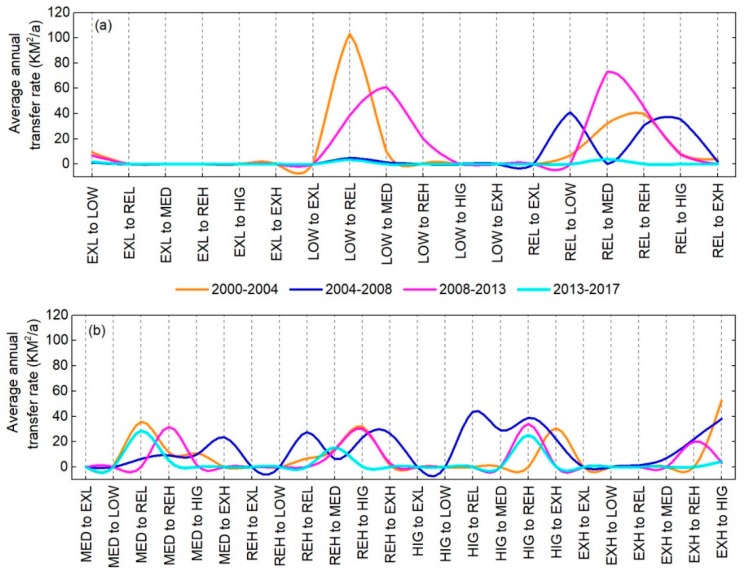
Annual transfer rates of different ecological risk grades during 2000–2014, 2004–2008, 2008–2013, and 2013–2017 (km^2^/year). (**a**) Map of annual transfer rates of different ecological risk grades transposed from EXL, LOW, and REL; (**b**) map of annual transfer rates of different ecological risk grades transposed from MED, REH, HIG, and EXH. Notes: EXL: extremely low; LOW: low; REL: relatively low; MED: medium, REH: relatively high; HIG: high; EXH: extremely high.

To better analyze the transformation relationship between landscape ecological risk areas of open coastal wetlands in Jiangsu (OCWJ) in different periods, an ecological risk grade transfer matrix was used to study the changes in the areas of ecological risk area at each grade. ArcGIS10.3 was used to superimpose the ecological risk grade areas of different periods, and the results of grade area conversion of ecological risk areas of the OCWJ during 2000–2017 were analyzed (Table 4 and Figure 9).

Overall, the change in landscape ecological risk levels in the OCWJ from 2000–2017 is substantial, and the areas of low, relatively low, extremely low, and medium ecological risk have all undergone large changes. The total area of transformation from low-grade to high-grade ecological risk is approximately 1910.71 km^2^, accounting for 71.75% of the total area converted. The total level of ecological risk from high-grade to low-grade is about 752.23 km^2^, accounting for 28.25% of the total study area. The area converted from low-grade to high-grade ecological risk is 2.54 times that of the area converted from high-grade to low-grade, indicating that the ecological risk in the study area has increased. Moreover, the most significant areas of ecological risk grade area conversion are those converted from low to medium ecological risk and from low to relatively high ecological risk, with conversion areas of 331.74 km^2^ and 273.45 km^2^, respectively. These conversions mainly occur in areas where the landscape type conversion is relatively large (e.g., the southeast coastal area of Sheyang, Dafeng and Dongtai), where human activities are pronounced. The MMLL related to fisheries is constantly expanding and the ecological risk is increasing.

A comparison of the average annual conversion rate of ecological risk levels of the OCWJ for different time periods is illustrated in Figure 9. It is evident that in 2000–2004, 2004–2008, 2008–2013, and 2013–2017 the annual average conversion rate from high-grade to low-grade ecological risk (i.e., LOW-EXL, REL-EXL, REL-LOW, MED-EXL, MED-LOW, MED-REL, REH-EXL, REH-LOW, REH-REL, REH-MED, HIG-EXL, HIG-LOW, HIG-REL, HIG-MED, HIG-REH, EXH-EXL, EXH-LOW, EXH-REL, EXH-MED, EXH-REH, and HIG-REH) exhibited a decreasing trend. Extremely high to HIG changed the most, and the average annual transfer rate decreased by 48.37 km^2^/year. In contrast, the average annual conversion rate of ecological risk level from low-grade to high-grade (i.e., EXL-LOW, EXL-REL, EXL-MED, EXL-REH, EXL-HIG, EXL-EXH, LOW-REL, LOW-MED, LOW-REH, LOW-HIG, LOW-EXH, REL-MED, REL-REH, REL-HIG, REL-EXH, MED-REH, MED-HIG, MED-EXH, REH-HIG, REH-EXH, and HIG-EXH) exhibited a fluctuating but increasing trend. The average annual conversion rate from LOW-REL reached 103.17 km^2^/year during 2000–2004. The average annual conversions rates from LOW-MED and from REL-MED were 60.85 km^2^/year and 73.01 km^2^/year, respectively, during 2008–2013. However, the average annual conversion rate from 2013–2017 was generally low, indicating that impact of human activities and occupancy on the coastal zone will gradually decrease in the future. In general, the expansion of ecological risk levels from low-grade to high-grade areas increases the level of ecological risk in the OCWJ, and thus it is necessary to design and implement rational land use planning strategies and policies to protect the ecological environment.

## 4. Discussion

Remote sensing techniques (RS) and geographic information systems (GIS) are an effective means of characterizing the evolution of landscape ecological risk patterns. However, an important problem is how to develop evolution models of landscape ecological risk patterns (LERP) with an optimal scale, auxiliary landscape metrics, and geographic information technology. We used appropriate grain and extent factors to select a multi-metrics optimal scale method (MMOS) for decreasing scale effect, to determine the appropriate combinations of landscape ecological risk indexes for constructing optimal LERP models in the OCWJ region. We found that human activities have significantly impacted the coastal landscape (Figure 4) and the pattern of evolution of ecological risk (Figure 7). These findings extend those of previous assessments of ecological risk on a regional scale and they provide an additional reference study for studies of changes in coastal zones worldwide.

Landscape ecological risk assessment (LERA) is useful for representing landscape ecological security, which is a critical domain of landscape sustainability [57]. The construction of an ecological risk model is based on the following considerations. First, the determination of indicators in the ecological risk model of this study focuses on the characterization of landscape disturbances or risk exposure process, highlighting the geographical-ecological significance of the indicators. Second, more indicators are derived from remote sensing data or model-based calculations, which enhance the objectivity and accuracy of the landscape ecological functions reflected by the indicators. Third, the introduction of spatial analysis embodies the geographical research concept of time conversion space, enriches the landscape ecological risk assessment system, and embodies the dynamic concept of ecological sustainability. Therefore, the landscape ecological risk assessment model based on landscape pattern indexes minimizes the subjective cognitive uncertainty of the researcher and the sensitivity of the indicators to change. Previous work has documented the effectiveness of LERA in assessing the quality of the eco-environment of the same area [17,55]. However, these studies have either been of areas other than coastal zones, or they have not considered the evolution of the spatial characteristics of landscape and landscape ecological risk at optimal scales. We compared our LERP models results with conventional analyses of the eco-environment of the same area [24,27,58], and the results indicate that in terms of ecological significance, our LERP models have a similar or better performance for observing landscape ecological risk patterns. Consequently, a LERP model together with the MMOS method is a cost-effective means of assessing landscape transformation risk in open coastal wetlands, and our results demonstrate the effectiveness of this approach for studying the long-term effects of anthropogenic activities in the coastal zone.

In principle, LERA based on spatial patterns is suitable for all types of risk integrative assessment, but it may not apply to those assessments that emphasize a specific process. The source–sink landscape model that seeks a quantitative expression of the coupling of the landscape pattern and ecological processes is an effective way of integrating patterns and processes [59]. Source-sink landscape types and risk processes can avoid the drawbacks of the LERA method that only relies on landscape pattern indexes. Therefore, risk assessment based on the source-sink landscape model is an important trend in future use of the LERP model. However, for different types of risk stress, the contributions from the source-sink landscape can even be transformed into one other, which increases the quantitative difficulty of the evaluation. Therefore, the risk peeling of the dominant ecological processes on the landscape scale, and the correct identification of the source and sink landscape relationships, are the main difficulties in applying the source landscape model to solve practical problems [60].

Our study also has several limitations that should be addressed in future research. First, the method of weight assignment largely depends on our empirical statistical work and the analysis of macro risk factors. Theoretical explanations of specific scale effects cannot be fully determined and need to be further investigated. Second, only spatial patterns were addressed in our study, with the neglect of the possible effects of stress specific processes; in particular, a means of integrating landscape patterns and ecological factors is needed. In fact, source-sink landscape types, especially risky process variables, have great potential for improving landscape ecological risk assessment models [20]. We intend to conduct additional research on the more specific risks of stress and the role of the object in our landscape ecological risk assessment models. Third, due to the long-term development and utilization of human beings and the fragmentation of habitats, many landscapes have been severely damaged. The landscape types are both intermittent and connected in a spatial distribution, and species diversity becomes a major attribute of this connection [5]. Therefore, by studying the landscape patterns characteristics and the evolution of ecological risks, the differences between species diversity of landscape types, the relationship with environmental factors, and their spatial distribution patterns can be revealed to some extent. In addition, the limitations of the research data acquisition, a more detailed analysis of species biodiversity content cannot be conducted. In future work we will explore whether changes in landscape pattern diversity and species diversity can be detected with the combination of higher resolution images and vegetation information. Fourth, in future investigations the division of ecological risk units needs to pay more attention to the integrated geographical significance of the evaluation units to promote the application of the risk assessment LERP models in other coastal areas.

## 5. Conclusions

We have constructed an optimal scale method with an appropriate extent and grain using multi-temporal Landsat TM/OLI images acquired in the years 2000, 2004, 2008, 2013, and 2017. We then developed landscape ecological risk indices. Based on this model, the entire process of spatiotemporal evolution of ecological risk patterns of open coastal wetlands in Jiangsu (OCWJ) was mapped and analyzed. Our main conclusions are as follows: The main landscape types in the study area are tidal flats and farmland, and the main features of the landscape evolution are that the area of aquafarming has increased substantially at the expense of the tidal flat area, and the landscape heterogeneity has become more pronounced. The heterogeneity of the landscape in the OCWJ area is mainly due to human disturbance and the impact of landscape reconstruction on the coastal wetland ecosystem. In the past 20 years, the areas of low and relatively low ecological risk in the study area have been greatly reduced and the areas of medium, relatively high and high ecological risk have greatly increased. The areas of high-grade ecological risk areas are mainly distributed in Dongtai and in the area of Dafeng. The area transformed from low-grade to high-grade ecological risk accounted for 71.75% of the study area during period 2000–2017. In the previous periods (2000–2004 and 2004–2008), the area of low-grade ecological risk was transformed to middle-grade ecological risk, and in the later periods (2008–2013 and 2013–2017) there was an increase in the proportion of ecological risk areas which were transformed to areas of high-grade ecological risk.

In general, reducing the ecological risk protects the original natural landscape, and a reduction in the rate of increase of man-made landform landscapes (MMLL) is crucial. The results of this study potentially provide important insights and data for coastal landscape planners, researchers, and managers; for linking satellite data with relevant policy formulation; for enhancing the official database of coastal landscape planning; and for assessing the potential effects of the MMLL process on coastal environments. They also provide an important reference for studies of change in coastal zones worldwide.

## Figures and Tables

**Table 1:** Details of the landsat remote sensing data used in this study (spatial resolution: 30 m).

Satellite	Sensors	Path/Row	Date	Satellite	Sensors	Path/Row	Date
Landsat 5	TM	120/36	13 December 2000	Landsat 5	TM	119/37	06 December 2000
Landsat 5	TM	120/36	08 December 2004	Landsat 5	TM	119/37	15 November 2004
Landsat 5	TM	120/36	19 December 2008	Landsat 5	TM	119/37	13 January 2009
Landsat 8	OLI	120/36	01 December 2013	Landsat 8	OLI	119/37	10 December 2013
Landsat 8	OLI	120/36	06 December 2017	Landsat 8	OLI	119/37	22 December 2017

**Table 2:** Commonly-used landscape metrics.

Level	Landscape Metric	Significance/Description	Themes/Method
Class	Number of patches (NP)	Total number of patches in the landscape	^a^ Measure of configuration/standard method
	Landscape shape index (LSI)	Landscape boundary and total edge within the landscape divided by the total area	^a/b^ Measure of configuration/standard method
	Landscape division index (DIVISION)	Degree of plaque fragmentation in the landscape, describing the complexity of the landscape	^a^ Diversity assessment/standard method
	Shannon’s diversity index (SHDI)	Heterogeneity of the landscape: the higher the landscape heterogeneity, the higher the diversity index	^a^ Diversity assessment/standard method
	Percentage of the landscape (PLAND)	Percentage of the landscape comprising of the corresponding patch type	^b^ Measure of configuration/standard method
	Patch density (PD)	Configuration metrics patch density	^b^ Spatial differentiation/standard method
	Total Area (CA)	Sum of the areas of all patches belonging to a given class	^b^ Measure of configuration/standard method
	Largest patch index (LPI)	Percentage of the landscape comprised by the largest patch of the corresponding patch type	^b^ Measure of configuration/standard method
	Edge Density (ED)	Amount of edge relative to the landscape area	^b^ Spatial differentiation/standard method
landscape	Patch Cohesion Index (COHESION)	Measures the physical connectedness of the corresponding patch type	^d^ Measure of configuration/moving window
	Euclidean nearest neighbor distance mean index (ENN–MN)	Distance to the nearest neighboring patch of the same type, based on shortest edge-to-edge distance	^d^ Measure of configuration /moving window
Class/landscape	Shannon’s evenness index (SHEI)	Expresses the uniformity of distribution at different landscape levels	^a/c^ Measure of configuration/standard method and moving window
	Contagion index (CONTAG)	Measures the extent to which patch types are aggregated or clumped	^a/c^ Measure of configuration/moving window

Note: ^a^ applicable to grain and extent; ^b^ applicable to overall landscape pattern characteristics; ^c^ applicable to block to base ratio; ^d^ applicable to landscape heterogeneity.

**Table 3:** Summary of landscape pattern indices for the study area.

Year	Types	SEA	TID	SAL	FAR	AQU	DRY	BUI	REE	SEE	COR
2000	*Fragmentation_i_*	0.04	0.01	0.01	0.00	0.01	0.01	0.00	0.05	0.13	0.01
	*Isolation_i_*	0.41	0.08	0.05	0.03	0.11	0.29	0.00	0.51	1.04	0.28
	*Dominant_i_*	2.13	8.37	3.28	9.40	3.50	0.75	0.00	1.62	1.12	1.39
	*Disturbance_i_*	0.57	1.70	0.67	1.89	0.73	0.24	0.00	0.50	0.60	0.37
	*Loss_i_*	0.07	0.25	0.06	0.21	0.12	0.04	0.00	0.02	0.03	0.03
2004	*Fragmentation_i_*	0.03	0.01	0.00	0.00	0.00	0.01	0.01	0.06	0.16	0.01
	*Isolation_i_*	0.37	0.10	0.04	0.04	0.07	0.38	1.41	0.74	1.35	0.29
	*Dominant_i_*	2.08	7.19	2.75	9.91	5.87	0.49	0.06	0.95	0.86	1.39
	*Disturbance_i_*	0.55	1.47	0.56	2.00	1.20	0.22	0.44	0.44	0.66	0.37
	*Loss_i_*	0.07	0.21	0.05	0.22	0.20	0.04	0.01	0.02	0.04	0.03
2008	*Fragmentation_i_*	0.03	0.01	0.00	0.00	0.00	0.01	0.01	0.02	0.10	0.02
	*Isolation_i_*	0.33	0.13	0.04	0.03	0.06	0.21	0.45	0.41	1.03	0.30
	*Dominant_i_*	2.12	6.28	2.12	9.71	6.87	1.01	0.22	1.01	0.88	1.33
	*Disturbance_i_*	0.54	1.30	0.44	1.95	1.39	0.27	0.18	0.34	0.53	0.36
	*Loss_i_*	0.07	0.19	0.04	0.21	0.23	0.05	0.00	0.01	0.03	0.03
2013	*Fragmentation_i_*	0.03	0.01	0.00	0.00	0.00	0.01	0.00	0.03	0.10	0.02
	*Isolation_i_*	0.30	0.13	0.09	0.04	0.05	0.18	0.17	0.44	1.34	0.37
	*Dominant_i_*	2.47	4.90	1.39	10.34	7.61	1.03	0.67	1.27	0.52	1.35
	*Disturbance_i_*	0.60	1.02	0.31	2.08	1.54	0.26	0.19	0.40	0.55	0.39
	*Loss_i_*	0.08	0.15	0.03	0.23	0.25	0.05	0.00	0.02	0.03	0.03
2017	*Fragmentation_i_*	0.03	0.01	0.00	0.00	0.00	0.04	0.00	0.03	0.10	0.01
	*Isolation_i_*	0.30	0.12	0.11	0.04	0.05	0.45	0.22	0.45	1.42	0.27
	*Dominant_i_*	2.37	4.26	1.28	10.23	7.81	1.74	0.67	1.31	0.52	1.51
	*Disturbance_i_*	0.58	0.89	0.29	2.06	1.58	0.50	0.20	0.41	0.58	0.39
	*Loss_i_*	0.07	0.13	0.03	0.22	0.26	0.09	0.00	0.02	0.03	0.03

Notes: *Fragility_i_* is the landscape fragility index; the values are as follows: SEA: seawater (0.13), TID: tidalflat (0.15), SAL: saltwork (0.09), FAR: farmland (0.11), AQU: aquafarm (0.16), DRY: drypool (0.18), BUI: building (0.02), REE: reedbed (0.04), SEE: seepweed (0.06), COR: cordgrass (0.07).

**Table 4:** Transition matrix for changes in the area (km^2^) of landscape types in the OCWJ during 2000–2017.

	Year 2017	EXL	LOW	REL	MED	REH	HIG	EXH	TOT
Year 2000									
EXL		483.71	66.12	17.59	0	0	0	0	567.42
LOW		1.63	6.98	124.74	331.74	273.45	88.15	0	826.68
REL		0	0	70.72	108.82	262.80	144.20	23.22	609.75
MED		0	0	1.56	36.99	119.01	99.83	10.03	267.42
REH		0	0	26.05	18.48	59.85	121.29	92.01	317.68
HIG		0	0	112.13	67.17	164.55	71.02	27.72	442.60
EXH		0	0	37.74	132.85	103.64	86.44	2.78	363.44
TOT		485.34	73.10	390.52	696.05	983.29	610.93	155.76	3394.98

Notes: EXL: extremely low; LOW: low; REL: relatively low; MED: medium, REH: relatively high; HIG: high; EXH: extremely high; TOT: total.
